# The complete mitochondrial genome of the Luhua chicken (Gallus gallus)

**DOI:** 10.1080/23802359.2020.1791021

**Published:** 2020-07-15

**Authors:** Jingjing Gu, Sheng Li

**Affiliations:** a College of Animal Science and Technology, Hunan Agricultural University, Changsha, China; b Hunan Key Laboratory for Genetic Improvement of Animals, Changsha, China; c Hunan Engineering Research Center of Poultry Production Safety, Changsha, China; d Maxun Biotechnology Institute, Changsha, China

**Keywords:** The Luhua chicken, mitochondrial genome, next-generation sequencing

## Abstract

The Luhua chicken is one of the excellent Chinese local chicken breeds. The first complete mitochondrial genome of Luhua chicken was assembled by using next generation sequencing method. The complete mitogenome contains one control region, 2 ribosomal RNAs, 13 protein-coding genes and 22 transfer RNA genes. This work provides a valuable resource for the mitochondrial research and contributes to genetic improvement of domestic chicken.

Luhua chicken, raised mainly in Southwest Shandong and surrounding provinces, is one of the excellent Chinese local chicken breeds. This chicken has a unique feather color pattern which is black and white feather with transverse spots covered all over the body. This breed is also known for its good meat quality and egg laying performance. To better understand the genetic background and to improve the breeding scheme for the future of the Luhua chicken, we collected a purebred individual from the central breeding area Wenshang county (35.73 N and 116.48 E), Shandong province, China. The Luhua chicken specimen (Voucher No. LH150879) was stored at −80 °C in the Museum of Hunan provincial key laboratory for genetic improvement of domestic animal, Changsha, China. This muscle specimen was used to extract the total genomic DNA. Illumina libraries were generated from the genomic DNA and sequenced on Illumina Hiseq 2500 sequencer. In total, we obtained 12.78 Gb raw sequence data and the sequences have been deposited in the NCBI Sequence Read Archive (SRA) with accession number SRR4302053. The complete mitochondrial genome of Luhua chicken was assembled and reported for the first time (GenBank accession number MT555049). We used tRNAscan-SE 2.0 (Chan and Lowe 2019) and MITOS (Bernt et al. 2013) to fully annotate the mitogenome sequences of Luhua chicken. The neighbor-joining (NJ) phylogenetic tree was constructed using Mega 7.0 (Kumar et al. 2016) with 1000 bootstrap replicates.

The complete mitochondrial genome sequence of Luhua chicken is a circular double-strand DNA molecule of 16,784 bp in length, containing 22 transfer RNA genes (tRNAs), 13 protein-coding genes (PCGs), 2 ribosomal RNA genes (rRNAs), and 1 non-coding region (D-loop). The overall base composition of the mitogenome is 30.26% A, 32.51% C, 23.71% T, and 13.51% G with biased toward A + T nucleotides (53.97%). The length of coding region is 15,553 bp. Most PCGs (12 out of 13), 14 tRNAs and 2 rRNAs are encoded on the heavy chain, while only one PCG (*ND6*) and 8 tRNAs are encoded on the light chain. The initiation codon of 12PCGs is ATG except for *COX1* initiated with GTG. There have four types of termination codon which are TAA, TAG, AGG and an incomplete termination codon T, which is the 5′ terminal of adjacent gene (Anderson et al. 1981). All these genes have 17 spaces and 10 overlaps. The 22 tRNA genes are scattered among rRNAs and PCGs, ranging from 66 to 76 bp in length.

The NJ tree was constructed using the whole mitochondrial genome of Luhua chicken and other 36 chickens retrieved from Genbank. The result (Figure 1) shows Luhua chicken is close related with Xuefeng, Nandan, Daweishan Mini, Xianju and Tibetan. However, Luhua chicken has the farthest genetics distance with Huang Lang. This work provides a valuable resource for the mitochondrial research and contributes to genetic improvement of domestic chicken.

**Figure 1.**
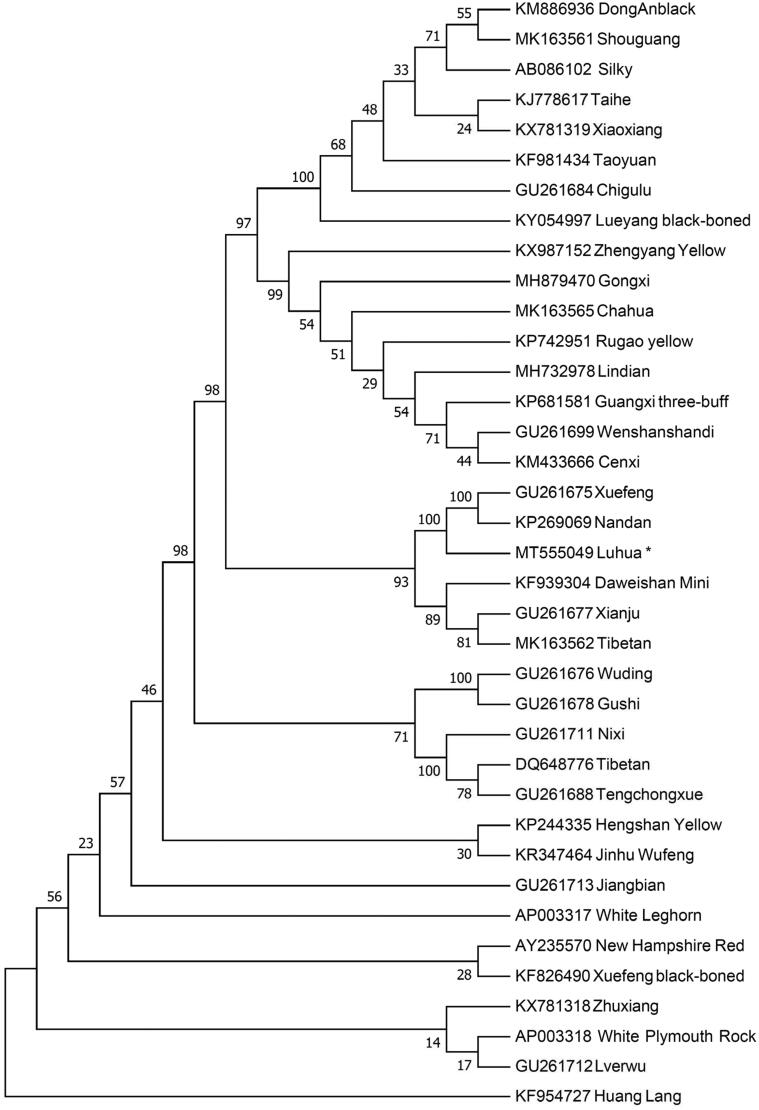
Neighbor-joining tree based on the complete mitochondrial DNA sequence of 37 chicken breeds. GenBank accession numbers are given before the species name.

## Data Availability

The sequence data that support the findings of this study are openly available in the NCBI Sequence Read Archive (SRA) at http://www.ncbi.nlm.nih.gov/sra/ with accession number SRR4302053. The complete mitochondrial genome of Luhua chicken (Gallus gallus) is openly available in GenBank at http://www.ncbi.nlm.nih.gov/genbank with accession number MT555049.
